# Prevalence and characteristics for mental health disorder before and after childhood cancer diagnosis—a statewide population-based study among Medicaid beneficiaries

**DOI:** 10.3389/fpsyg.2025.1680382

**Published:** 2026-02-04

**Authors:** Feitong Lei, Laurie E. McLouth, Eric B. Durbin, Thomas T. Tucker, John L. Villano, Bin Huang

**Affiliations:** 1 Division of Cancer Biostatistics, Department of Internal Medicine, College of Medicine, University of Kentucky, Lexington, KY, United States; 2 Department of Behavioral Science, Center for Health Equity Transformation, College of Medicine, University of Kentucky, Lexington, KY, United States; 3 Kentucky Cancer Registry, Markey Cancer Center, University of Kentucky, Lexington, KY, United States; 4 Division of Biomedical Informatics, Department of Internal Medicine, College of Medicine, University of Kentucky, Lexington, KY, United States; 5 Department of Epidemiology, College of Public Health, University of Kentucky, Lexington, KY, United States; 6 Clinical Neuro-Oncology Research Program, Department of Internal Medicine, University of Kentucky Medical Center, Lexington, KY, United States

**Keywords:** childhood cancer patients, Medicaid, mental health disorder, population-based, prevalence

## Abstract

**Introduction:**

Pediatric cancer patients suffer mental health deficits. Patients who are vulnerable with respect to socioeconomic or other sociodemographic factors may be at heightened risk for worse mental health outcomes during cancer for many reasons including entering treatment with an existing mental health disorder. The purpose of this study was to describe the prevalence and trajectory of mental health before and after a childhood cancer diagnosis in a socio-demographically at-risk sample.

**Methods:**

Data from the Kentucky Cancer Registry was utilized to identify patients aged 19 or under with a first primary childhood cancer diagnosis during 2001–2017. Linking KCR data with Medicaid claims, we included patients with continuous Medicaid enrollment 12 months before and after their cancer diagnosis. MHDs were identified using both International Classification of Diseases (ICD)-9 and ICD-10 diagnosis codes in Medicaid claims.

**Results:**

Of the 978 patients, 54% were male, and 39% were from Appalachian counties. The most common cancers diagnosed were leukemias (*n* = 238), brain and central nervous system (*n* = 220), and lymphomas (*n* = 147). For the 12-month pre-cancer diagnosis period, 32% (*n* = 310) of the patients had a MHD, increasing to 55% (*n* = 540) in the 12 months post-diagnosis period. The most frequent MHDs were mood disorder (before *n* = 120; after *n* = 311) and neuropsychiatric/developmental disorders (before *n* = 228; after *n* = 267). Mood disorders increased from 12% pre-cancer diagnosis to 32% post-cancer diagnosis, from 10 to 37% for lymphoma patients, and from 15 to 64% for bone cancer patients.

**Conclusion:**

Over half of the Medicaid-enrolled childhood cancer patients in Kentucky diagnosed with MHDs within a year of their cancer diagnosis, with a notable increase from pre-diagnosis levels. This increased prevalence post-diagnosis may result from the identification of pre-existing MHDs during cancer treatment, or the emergence of new MHD because of the cancer diagnosis and treatment. Our study highlights the psychosocial needs that extend beyond standard cancer treatment and underscores the importance of psychosocial services during and after the cancer treatment period.

## 1 Introduction

An estimated 496,000 childhood cancers survivors live in the United States (US) as of 2020 (National Cancer Institution, 2023). With improved cancer treatments, the 5-year survival of childhood cancers has increased to 85% in recent years, underscoring the critical importance of maximizing their long-term health and quality of life (National Cancer Institution, 2023). Unfortunately, many childhood cancer survivors suffer from mental health disorders (MHDs) during and after cancer treatment, with negative effects on cognitive functioning, quality of life, and social relationships (Stuber et al., 2010). Cancer diagnosis and treatment are inherently stressful experiences for childhood cancer patients and their families. The intense treatment regimens, which often include chemotherapy, radiation, and prolonged hospital stays, disrupt patients' normal routines, leading to feelings of isolation, fear, anxiety, and uncertainty (Marusak et al., 2018; Jibb et al., 2022; Wakefield et al., 2010). Such experiences can further manifest in MHDs. Large-scale systematic reviews demonstrate that cancer survivors experience substantially higher rates of depression, anxiety, and posttraumatic stress disorder than their non-cancer peers (Marchak et al., 2022). While the need for psychosocial service is clear, 49% children with a MHD never receive it, mirroring the poor psychosocial service provision seen in their unaffected peers who suffer from a mental health concern in the U.S. (Whitney and Peterson, 2019).

Medicaid beneficiaries represent a substantial and growing share of the childhood cancer patients. A study reported that in 2015, about 40% of children aged 0–15 with cancer were enrolled in Medicaid nationwide, a proportion expected to rise with Medicaid expansion in more states (Barnes et al., 2020). Kentucky implemented Medicaid expansion on January 1, 2014, under the Affordable Care Act, and as of mid-2025, the Kentucky Medicaid program provides insurance to approximately 1.4 million residents, making it a critical case for examining MHD among publicly insured childhood cancer patients (Centers for Medicare Medicaid Services, 2025). Unfortunately, many of the mental health inequities due to socioeconomic seen in the adult cancer population are likely to also affect childhood cancer survivors. Mental health outcomes are notably worse in underserved populations, including those who are low-income, racial or ethnic minorities, or geographically isolated (Mongelli et al., 2020; Knifton and Inglis, 2020). Childhood cancer survivors in rural or socioeconomically disadvantaged areas often have limited access to psychosocial service and social support systems, even though when insured through Medicaid (Andrilla et al., 2018). In the broader pediatric population, nearly 30% of publicly insured children in 2021 had a mental, emotional, or developmental condition—higher than privately insured peers (22%) (Centers for Medicare Medicaid Services, 2023). Despite high need, Medicaid-insured children frequently experience delays in diagnosis and reduced access to mental health services. The disparities can lead to a higher prevalence of untreated or poorly managed MHDs in these populations. Moreover, many of these survivors face numerous socioecological risks that heighten their vulnerability to developing MHDs.

However, most existing research on mental health outcomes in childhood cancer patients or survivors, including the Childhood Cancer Survivor Study, is drawn from cohorts treated at tertiary cancer centers and may not represent the general background population or Medicaid population (Ness et al., 2009). Additionally, few longitudinal studies have assessed mental health both before and after cancer diagnosis to establish baseline rates and trajectories, as most assess mental health outcomes only during survivorship (Marchak et al., 2022). While administrative claims data have limitations, they provide unique opportunities for population-based research on real-world psychosocial service utilization, capturing all medically diagnosed MHDs across entire state programs. Given that Medicaid expansion has increased coverage more substantially among population from underrepresented backgrounds (Lin et al., 2021), studies using Medicaid administrative data are essential to understanding mental health disparities in this vulnerable population.

Understanding the prevalence and trajectory of MHDs of childhood cancer survivors is critical to identifying those at higher risk for poor outcomes and determining optimal intervention points. To date, population-based studies providing comprehensive, representative insights into the prevalence of MHDs among Medicaid-enrolled childhood cancer survivors have been lacking. This study addresses this gap by using statewide data linked with Kentucky Medicaid claims to examine MHD prevalence among childhood cancer patients before and after diagnosis. Additionally, we examine variations in MHD prevalence across demographic groups and cancer sites to better understand disparities within this vulnerable population.

## 2 Material and methods

### 2.1 Data source

We utilized data from the Kentucky Cancer Registry (KCR) to identify childhood cancer patients diagnosed from 2001 to 2017. The KCR is a population-based state cancer registry that is supported by the National Cancer Institute's Surveillance, Epidemiology, and End Results (SEER) Program and the Centers for Disease Control and Prevention's National Program of Cancer Registries. The KCR provides data on patient demographics, tumor characteristics, first-course treatment, and survival outcomes. In this study, probabilistic methods were used to link KCR data with Medicaid claims records (Gallaway et al., 2019). Medicaid data includes beneficiaries' healthcare service records from the time of insurance eligibility until dropout or death. The dataset includes inpatient claims, outpatient claims, and prescription drug use. Additionally, to obtain county-level socioeconomic information associated with each patient, KCR data were linked with the American Community Survey (2007–2011) based on patients' county of residence at diagnosis (United States Census Bureau, 2024).

### 2.2 Study population

KCR data was used to identify childhood cancer patients aged 19 or younger with a first primary cancer diagnosis in Kentucky between 2001 and 2017. To reduce potential bias from patients with missing claims and to ensure comprehensive patient information before and after cancer diagnosis, patients without continuous Medicaid enrollment for 12 months before and 12 months after the cancer diagnosis were excluded. Patients who died within the first year after diagnosis were included if they maintained continuous Medicaid coverage until death.

### 2.3 Mental health disorders (MHDs)

To comprehensively identify MHD diagnoses within the Medicaid claims data, we utilized the Child and Adolescent Mental Health Disorders Classification System (CAMHD-CS; Zima et al., 2020). The CAMHD-CS maps both International Classification of Diseases, 9th Revision (ICD-9), and 10th Revision (ICD-10) codes to Diagnostic and Statistical Manual of Mental Disorders, Fifth Edition (DSM-5) diagnoses, categorizing them into 30 MHD groups. In this study, patients with any diagnostic code (whether primary or additional diagnoses) included in the CAMHD-CS were identified as having experienced an MHD. To streamline the analysis, we consolidated the 30 MHD diagnoses into seven broad categories, based on a crosswalk between the International Classification of Diseases, Ninth Revision (ICD-9) and Tenth Revision (ICD-10) codes, and the classification framework used in (Hovén et al., 2020) study. Categories included psychotic disorders, mood disorders, personality disorders, substance use disorders, neuropsychiatric/developmental disorders, eating disorders, and other MHDs. Details on how the 30 MHD groups and their corresponding ICD-9 and ICD-10 codes, as defined by the CAMHD-CS, were classified into the seven MHD categories are presented in Supplementary Table S1. These categories were used to describe the pre-diagnosis MHD based on claims from the 12 months before the cancer diagnosis, and post-diagnosis MHD based on claims from the first 12 months after cancer diagnosis.

### 2.4 Demographic and clinical variables

Demographic and clinical variables in the analysis included: age at cancer diagnosis, gender, race (White, Black, or other), Appalachian residence (yes/no), residency metro status (metro/non-metro), quantile of county-level educational attainment, quantile of county-level percent below poverty, year of cancer diagnosis, and cancer sites. Age at cancer diagnosis was classified into four groups: 0–4, 5–9, 10–14, and 15–19 years. Appalachian residence was based on whether the patient's county of residence was defined as Appalachia by the Appalachian Regional Commission (2021). Residency metro status was based on 2013 Rual-Urban Continuum Codes (metro: 1–3; non-metro: 4–9). County level quantiles of education and poverty estimates were generated from the American Community Survey 2007–2011 and linked with the patients' county of residence at diagnosis. County educational attainment was defined as the proportion of individuals with high school or higher education. County poverty estimates were defined as the proportion of individuals living below the poverty level in the county. Year of diagnosis was used to define four time periods: 2001–2005, 2006–2009, 2010–2013, and 2014–2017. Childhood cancer sites were categorized into major groups defined by the third edition of the International Classification of Childhood Cancer (ICCC-3; Steliarova-Foucher et al., 2005).

### 2.5 Statistical analysis

Descriptive statistics were utilized to summarize categorical variables as counts and percentages for pre-diagnosis and post-diagnosis MHDs. Differences in the prevalence of MHDs across various demographic and clinical groups were assessed using the chi-square test.

To identify associated factors for MHD diagnoses, univariate logistic regression analysis was conducted. Factors that were statistically significant in the univariable analysis were subsequently included in a multivariable logistic regression model to adjust for potential confounders. The final model included only significant variables based on a backward elimination approach. Goodness of Fit measures were examined.

Since mood disorders and neuropsychiatric/developmental disorders were the most prevalent mental health diagnoses both before and after cancer diagnosis, subgroup analyses were performed on these two MHD categories, following the same procedures as the primary analyses. Statistical significance was defined as a two-sided *p*-value of less than 0.05. All statistical analyses were performed using SAS software, version 9.4 (SAS Institute Inc., Cary, NC).

## 3 Results

We identified 3,765 childhood cancer patients diagnosed between 2001 and 2017 from the KCR. Of these, 1,717 (46%) had evidence of Medicaid enrollment within 12 months of diagnosis. After excluding patients without continuous Medicaid in the 12 months before and after cancer diagnosis, the final study cohort included 978 eligible patients. In the study cohort, the median age was 9 years [interquartile range (IQR): 4–14]. As shown in Table 1, 31% (*n* = 306) of patients were in the 0–4 age group, 22% in the 5–9 age group, 25% in the 10–14 age group, and 31% in the 15–19 age group (Table 1). The cohort consisted of 54% males, 85% white, 55% residing in non-metro counties, and 39% residing in Appalachian counties. 330 (34%) patients were diagnosed in 2014–2017. The most prevalent cancers were leukemia (*n* = 238; 24%), brain and central nervous system (CNS) tumors (*n* = 220; 22%), and lymphoma (*n* = 147; 15%).

**Table 1:** Characteristics of Medicaid-enrolled childhood cancer patients diagnosed from 2001 to 2017 in Kentucky stratified by pre-diagnosis and post-diagnosis MHD status.

**Characteristic**	**Category**	**Total**	**With pre-diagnosis MHD**		***p*-value**	**With post-diagnosis MHD**		***p*-value**
			**No (*****n*** = **668)**	**Yes (*****n*** = **310)**		**No (*****n*** = **438)**	**Yes (*****n*** = **540)**	
			***N*** **(%)**	***N*** **(%)**	**(2 sided)**	***N*** **(%)**	***N*** **(%)**	**2 sided)**
Age group	0–4	306	239 (78.1)	67 (21.9)	< 0.0001	162 (52.9)	144 (47.1)	0.0007
	5–9	212	138 (65.1)	74 (34.9)		87 (41.0)	125 (59)	
	10–14	217	146 (67.3)	71 (32.7)		101 (46.5)	116 (53.5)	
	15–19	243	145 (59.7)	98 (40.3)		88 (36.2)	155 (63.8)	
Gender	Male	528	356 (67.4)	172 (32.6)	0.5225	226 (42.8)	302 (57.2)	0.1769
	Female	450	312 (69.3)	138 (30.7)		212 (47.1)	238 (52.9)	
Race	White	831	567 (68.2)	264 (31.8)	0.3606	367 (44.2)	464 (55.8)	0.3287
	Black	129	86 (66.7)	43 (33.3)		60 (46.5)	69 (53.5)	
	Other	18	15 (83.3)	3 (16.7)		11 (61.1)	7 (38.9)	
Metro status	Non-metro	534	373 (69.9)	161 (30.1)	0.254	249 (46.6)	285 (53.4)	0.2035
	Metro	444	295 (66.4)	149 (33.6)		189 (42.6)	255 (57.4)	
Appalachian status	Non-Appalachia	593	402 (67.8)	191 (32.2)	0.6695	263 (44.4)	330 (55.6)	0.7345
	Appalachia	385	266 (69.1)	119 (30.9)		175 (45.5)	210 (54.5)	
% High school or higher education	0%−73.16%	251	176 (70.1)	75 (29.9)	0.4296	119 (47.4)	132 (52.6)	0.1751
	73.17%−81.86%	244	174 (71.3)	70 (28.7)		114 (46.7)	130 (53.3)	
	81.97%−87.95%	247	163 (66)	84 (34)		114 (46.2)	133 (53.8)	
	≥87.96%	236	155 (65.7)	81 (34.3)		91 (38.6)	145 (61.4)	
% Below poverty 2010	0%−16.45%	360	236 (65.6)	124 (34.4)	0.31	152 (42.2)	208 (57.8)	0.2594
	16.46%−18.91%	147	97 (66)	50 (34)		60 (40.8)	87 (59.2)	
	18.92%−23.67%	234	164 (70.1)	70 (29.9)		110 (47)	124 (53)	
	≥23.68%	237	171 (72.2)	66 (27.8)		116 (48.9)	121 (51.1)	
Year of diagnosis	2001–2005	198	142 (71.7)	56 (28.3)	0.6136	114 (57.6)	84 (42.4)	0.0001
	2006–2009	192	127 (66.1)	65 (33.9)		76 (39.6)	116 (60.4)	
	2010–2013	258	178 (69)	80 (31)		120 (46.5)	138 (53.5)	
	2014–2017	330	221 (67)	109 (33)		128 (38.8)	202 (61.2)	
ICCC site	Leukemias, myeloproliferative and myelodysplastic diseases	238	171 (71.8)	67 (28.2)	0.179	104 (43.7)	134 (56.3)	0.0281
	Lymphomas and reticuloendothelial neoplasms	147	97 (66)	50 (34)		63 (42.9)	84 (57.1)	
	CNS and miscellaneous intracranial and intraspinal neoplasms	220	139 (63.2)	81 (36.8)		91 (41.4)	129 (58.6)	
	Neuroblastoma and other peripheral nervous cell tumors	50	36 (72)	14 (28)		25 (50)	25 (50)	
	Retinoblastoma	14	12 (85.7)	2 (14.3)		10 (71.4)	4 (28.6)	
	Renal tumors	33	27 (81.8)	6 (18.2)		22 (66.7)	11 (33.3)	
	Hepatic tumors	11	7 (63.6)	4 (36.4)		4 (36.4)	7 (63.6)	
	Malignant bone tumors	58	38 (65.5)	20 (34.5)		17 (29.3)	41 (70.7)	
	Soft tissue and other extraosseous sarcomas	55	44 (80)	11 (20)		26 (47.3)	29 (52.7)	
	Germ cell tumors, trophoblastic tumors and neoplasms of gonads	49	29 (59.2)	20 (40.8)		27 (55.1)	22 (44.9)	
	Other malignant epithelial neoplasms and malignant melanomas	90	60 (66.7)	30 (33.3)		45 (50)	45 (50)	
	Other and unspecified malignant neoplasms	1	1 (100)	0 (0)		0 (0)	1 (100)	
	Unknown	12	7 (58.3)	5 (41.7)		4 (33.3)	8 (66.7)	
With pre-diagnosis MHD	No					377 (56.4)	291 (43.6)	< 0.0001
	Yes					61 (19.7)	249 (80.3)	

### 3.1 Pre-diagnosis MHDs

Pre-diagnosis MHDs were identified in 32% (*n* = 310; Table 1) of patients within 12 months before their cancer diagnosis. Age at diagnosis is associated with pre-diagnosis MHD, with patients aged 15–19 years having the highest prevalence (40.3%) compared to younger age groups (0–4 years, 21.9%; 5–9 years, 34.9%; 10–14 years, 32.7%). Gender and race did not show significant differences in pre-diagnosis MHD prevalence.

Metro and Appalachian residence did not significantly differ in pre-diagnosis MHD prevalence, with similar rates in metro (33.6%) and non-metro areas (30.1%), as well as in Appalachian (30.9%) and non-Appalachian regions (32.2%). Socio-economic indicators such as county high school completion rates and poverty levels showed no significant differences or discernible patterns in MHD distribution. As for the year of diagnosis and cancer sites, no significant associations with pre-diagnosis MHD prevalence were observed.

In the logistic regression, only the age at diagnosis was found to be significant with pre-diagnosis MHD in the univariate analysis. Children diagnosed with cancer at ages 5–9 (OR = 1.9, 95% CI: 1.3–2.8), 10–14 (OR = 1.7, 95% CI: 1.2–2.6), and 15–19 (OR = 2.4, 95% CI: 1.7–3.5) had higher odds of having a MHD diagnosis prior to cancer diagnosis compared to those diagnosed at ages 0–4 (Table 2).

**Table 2:** Multivariable logistic regression analysis for identifying risks factors for pre- and post-diagnosis MHD.

**Effect**	**Pre-diagnosis MHD**		**Post-diagnosis MHD**	
	**Odds ratio**	**95% CI**	**Odds ratio**	**95% CI**
**Age at diagnosis (reference: 0–4)**				
5–9	1.9	1.3–2.8	1.3	0.9–2.0
10–14	1.7	1.2–2.6	1.1	0.7–1.6
15–19	2.4	1.7–3.5	1.8	1.2–2.8
**Year of diagnosis (reference: 2014–2017)**				
2001–2005	–	–	0.4	0.3–0.6
2006–2009	–	–	0.9	0.6–1.3
2010–2013	–	–	0.7	0.5–0.9
**ICCC (reference: lymphomas and reticuloendothelial neoplasms)**				
CNS and miscellaneous intracranial and intraspinal neoplasms	–	–	1.2	0.7–1.9
Germ cell tumors, trophoblastic tumors and neoplasms of gonads	–	–	0.5	0.2–0.9
Hepatic tumors	–	–	1.6	0.4–6.4
Leukemias, myeloproliferative and myelodysplastic diseases	–	–	1.2	0.8–1.9
Malignant bone tumors	–	–	2.1	1.1–4.4
Neuroblastoma and other peripheral nervous cell tumors	–	–	1.1	0.5–2.3
Other and unspecified malignant neoplasms	–	–	NA^*^	NA^*^
Other malignant epithelial neoplasms and malignant melanomas	–	–	0.6	0.3–1.1
Renal tumors	–	–	0.5	0.1–1.6
Retinoblastoma	–	–	0.5	0.1–1.3
Soft tissue and other extraosseous sarcomas	–	–	1.0	0.5–2.0
Unknown	–	–	1.3	0.4–5.2
**Pre-diagnosis MHD (reference: no)**				
Yes	–	–	5.2	3.8–7.3

^*^NA indicates unstable estimates resulting from small sample sizes.

### 3.2 Post-diagnosis MHDs

The MHD prevalence increased to 55.2% (540 patients) within 12 months after a childhood cancer diagnosis. The post-diagnosis MHD prevalence varied among age groups, with the highest prevalence in teenagers (15–19 years, 63.8%; Table 1), followed by children ages 5–9 years (59%), 10–14 years (53.5%), and 0–4 years (47.1%). Gender and race differences were not significant.

Similar post-diagnosis MHD observed across metro (57.4%) and non-metro areas (53.4%), as well as Appalachian (54.5%) and non-Appalachian regions (55.6%). Socio-economic indicators such as county high school completion rates and poverty levels showed no significant differences. However, the year of diagnosis significantly impacted MHD prevalence, with the highest rates in the 2014–2017 period (61.2%). Regarding cancer sites, the highest prevalence of post-diagnosis MHD was in patients with malignant bone tumors (70.7%), hepatic tumors (63.6%), and CNS neoplasms (58.6%), while the lowest was in children with renal tumors (33.3%) and retinoblastoma (28.6%).

Univariate logistic regression analysis showed that age at diagnosis, diagnosis year, ICCC cancer sites, and pre-diagnosis MHD were associated with the odds of post-diagnosis MHD. The multivariate Logistic regression model shows that children diagnosed at 15–19 (OR = 1.8, 95% CI: 1.2–2.8) had higher odds of post-diagnosis MHD compared to those diagnosed at ages 0–4. Those diagnosed in 2001–2005 (OR = 0.4, 95% CI: 0.3–0.6) and 2010–2013 (OR = 0.7, 95% CI: 0.5–0.9) had lower odds of MHD compared to 2014–2017. Cancer sites are associated with the likelihood of post-diagnosis MHD as well (*p* = 0.027). For example, compared to Lymphoma, germ cell tumors (OR = 0.5, 95% CI: 0.2–0.9) showed lower odds of post-diagnosis MHD while malignant Bone Tumors shower higher odds (OR = 2.1, 95% CI: 1.1–4.4). The remaining sites showed similar odds of MHD as Lymphoma. Compared to those without, with pre-diagnosis MHD was associated with higher likelihood of post-diagnosis MHD (OR = 5.2, 95% CI: 3.8–7.3).

### 3.3 Pre- and post-diagnosis MHD change

**Figure 1:**
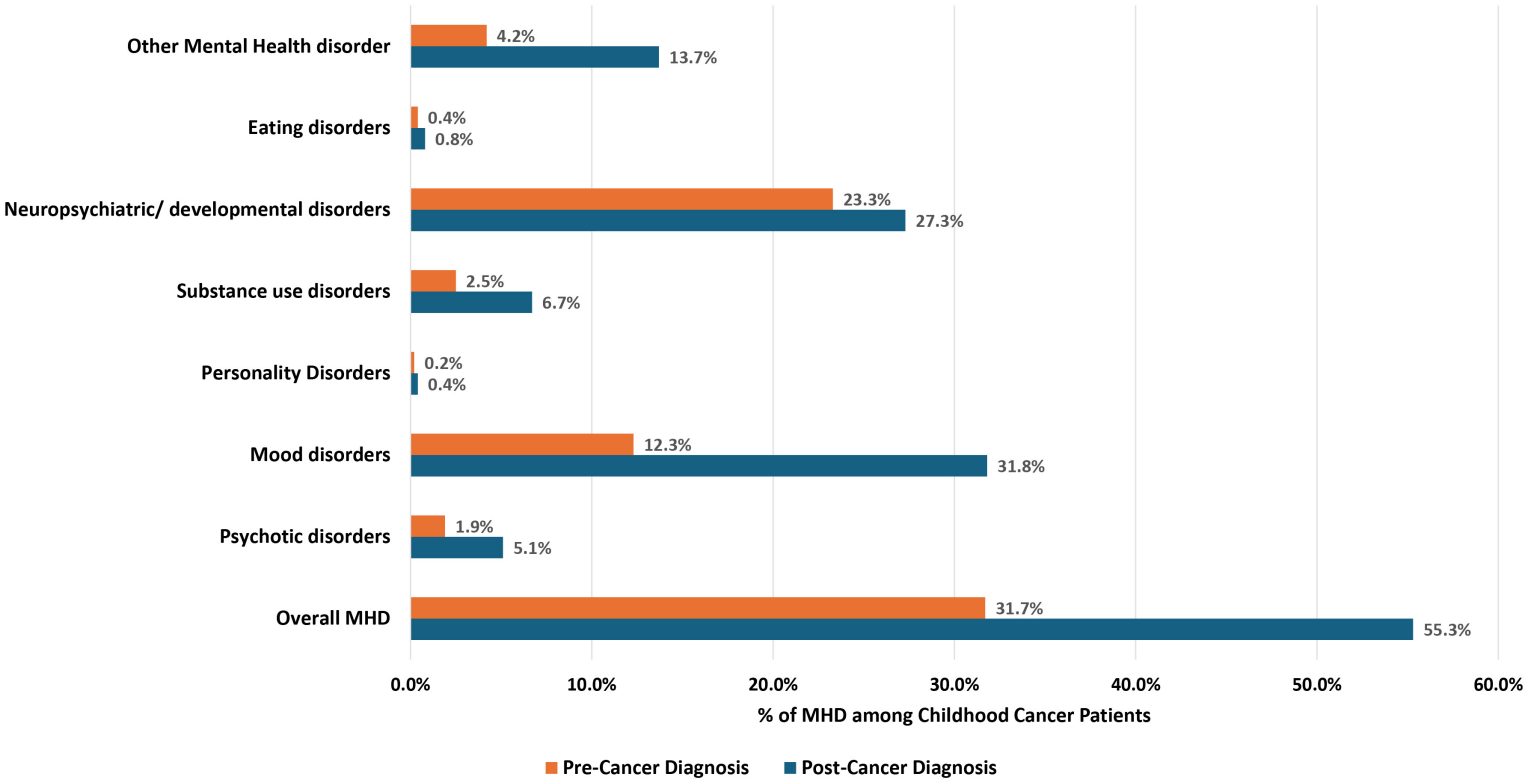
Percentage of MHD among childhood cancer patients in Kentucky, diagnosed from 2001 to 2017.

The distribution of MHD among childhood cancer survivors before and after diagnosis reveals several differences. The overall prevalence of MHD increased substantially post-diagnosis, rising from 31.7% to 55.2%. Substance use disorders increased from 2.5% to 6.7%, personality disorders rose marginally from 0.2% to 0.4%, and psychotic disorders saw an increase from 1.9% to 5.1%. Other MHDs showed a pronounced rise from 4.2% to 13.7% (Figure 1).

Certain subgroups exhibited notably higher increases in MHD prevalence. Age-wise, teenagers (15–19 years) showed an increase from 40.3% to 63.8%. Patients diagnosed in more recent periods, particularly from 2014 to 2017, experienced an increase from 33% to 61.2%. Additionally, specific cancer sites such as malignant bone tumors (*n* = 58) exhibited a substantial rise in MHD prevalence from 34.5% to 64.3%, and CNS neoplasms (*n* = 220) showed an increase from 36.8% to 58.6%.

### 3.4 Neuropsychiatric/developmental and mood disorders

Prior to cancer diagnosis, neuropsychiatric/developmental disorders (*n* = 228) and mood disorders (*n* = 120) were the most common MHDs, with depression occurring in 43% of mood disorder patients. Mood disorders (*n* = 311) and neuropsychiatric/developmental disorder (*n* = 267) were still the most common MHDs after cancer diagnosis. Neuropsychiatric/developmental disorders increased from 23.3% to 27.9%. Mood disorders rose markedly from 12.3% to 31.8%, depression disorders nearly doubled from 5.3% to 11.0%.

Children aged 15–19 years exhibited the highest prevalence of both pre-diagnosis (27.6%; Supplementary Table S2) and post-diagnosis (46.9%; Supplementary Table S2) mood disorders. Prevalence of mood disorders also showed significant differences across gender, region, cancer site, and year of diagnosis. Females had higher odds of pre-diagnosis mood disorders compared to males (OR = 1.6, 95% CI: 1.0–2.3; Supplementary Table S3), and white patients had higher odds of post-diagnosis mood disorders compared to black patients (OR = 1.8, 95% CI: 1.1–2.8). Those with malignant bone tumors had higher odds of post-diagnosis mood disorders compared to patients with leukemia (OR = 2.5, 95% CI: 1.3–4.9). Pre-diagnosis mood disorder associated with higher odds of post-diagnosis mood disorder (OR=5.6, 95% CI: 3.5–9.0).

In contrast, children aged 5–9 years showed the highest prevalence of neuropsychiatric/developmental both pre- (28.8%; Supplementary Table S4) and post-diagnosis (32.5%; Supplementary Table S4), with males having higher odds compared to females (e.g., OR = 1.5, 95% CI: 1.1–2.1 for pre- diagnosis; Supplementary Table S5). Children in metro areas had higher prevalence of both pre-diagnosis (26.8 vs. 20.4%) and post-diagnosis (31.3 vs. 24.0%) neurodevelopmental disorders compared to those in non-metro areas. Non-Appalachian residents showed a higher prevalence of pre-diagnosis neurodevelopmental disorders (26.1 vs. 19.0%) and post-diagnosis (29.3 vs. 24.2%). Moreover, areas with the lowest poverty levels had the highest prevalence of both pre-diagnosis (28.1%) and post-diagnosis (31.7%) neurodevelopmental disorders. Results from the logistic regression models showed that children from regions with lower poverty levels had significantly higher odds of pre-diagnosis neurodevelopmental disorders compared to those in higher-poverty regions (e.g., 0%−16.45% poverty, OR = 2.3, 95% CI: 1.5–3.5 vs. ≥23.68%). Additionally, children diagnosed in earlier periods (2006–2009) had higher odds of post-diagnosis neurodevelopmental disorders compared to more recent periods (2014–2017; OR = 1.6, 95% CI: 1.0–2.6). Pre-diagnosis neuropsychiatric/developmental disorder associated with increased odd of the post-diagnosis (OR = 15.2, 95% CI: 10.5–21.9).

## 4 Discussion

Our study is the first known to utilize statewide population-based data to examine the prevalence of MHDs in Medicaid-enrolled childhood cancer patients. We described the prevalence of MHDs among childhood cancer patients, with particular attention to the period before and after cancer diagnosis. Despite the importance of MHD among childhood cancer patients, research in this area remains limited-especially among underserved groups -such as Medicaid beneficiaries. By focusing on a Medicaid-enrolled cohort, our study specifically addresses a significant gap in the literature and highlights disparities among socioeconomically disadvantaged childhood cancer patients.

The findings of this study highlight the prevalence of MHDs among childhood cancer patients enrolled in Medicaid in Kentucky, with a marked increase in MHDs following cancer diagnosis. Notably, more than half (55%) of the patients experienced an MHD within 12 months after cancer diagnosis in our cohort, and about 32% of them were with mood disorders, such as anxiety, depression, and trauma and stressor-relate disorders. This aligns with prior studies that have documented substantial mental health burden among childhood cancer patients. Our observed prevalence falls within the ranges documented in the comprehensive review, reporting 2%−40% depression and other mood disorder across studies (Marchak et al., 2022). Similarly, another systematic reviews have reported pooled prevalence rates of 13.92% for anxiety, 20.43% for depression, and 20.90% for PTSD among children and adolescents with cancer (Al-Saadi et al., 2022). One study conducted in Poland reported higher prevalence, they found that 69% of children undergoing cancer treatment reported severe depression, while 61% experienced heightened anxiety (Lewandowska et al., 2021). Furthermore, compared to non-cancer controls, childhood cancer survivors are also at a higher risk of developing posttraumatic stress disorder (Stuber et al., 2010), with up to 20% still exhibiting symptoms years after completing treatment (Michel et al., 2019). Childhood, adolescent, and young adult cancer survivors were found to be 57% more likely to develop depression and 29% more likely to develop anxiety compared to healthy controls (Lee et al., 2023). The increased risk of suicidal ideation also reported among childhood cancer survivors, with higher rates of late [Odds Ratio (OR) =1.9] and recurrent suicide ideation (OR = 2.6) compared to siblings, which eventually led to worse survival [Hazard Ratio (HR) = 1.3] (Brinkman et al., 2014). Furthermore, the 55% prevalence of MHDs within 12 months post-diagnosis observed in our Medicaid-enrolled childhood cancer patients cohort is notably higher than the 30% prevalence of mental, emotional, or developmental conditions reported among publicly insured children in the general population (Centers for Medicare Medicaid Services, 2023), underscoring the particularly elevated mental health burden experienced by pediatric cancer patients. This elevated prevalence potentially reflects both the unique psychological impact of cancer diagnosis and treatment, as well as the compounding vulnerabilities of this population, including socioeconomic challenges, limited access to mental health services, and systemic barriers to care that disproportionately affect Medicaid beneficiaries.

This high burden and increase in MHDs from pre- to post-diagnosis is consistent with previous research, which has reported a similar pattern, particularly for mood disorders (Langeveld et al., 2002; Neu et al., 2021; Papini et al., 2023; Nathan et al., 2018; Zeltzer et al., 2009; Osmani et al., 2023; Lu et al., 2016). There is limited research comparing MHD before and after a cancer diagnosis among childhood cancer patients; thus, we reference a study on adult cancer patients for comparison. The study found that adult cancer patients face heightened risks of developing common MHDs, starting even a year before their cancer diagnosis. Notably, the relative rate of MHDs began rising 10 months before diagnosis (HR, 1.1), peaked in the first week post-diagnosis (HR, 6.7), and remained slightly elevated even a decade later (HR, 1.1) compared to cancer-free controls (Lu et al., 2016). This increase can be attributed to many factors. The psychological stress of the cancer diagnosis itself can profoundly impact a child's mental health, triggering anxiety and depression (Liu et al., 2023; Fortin et al., 2021; Friend et al., 2018). The isolation process of cancer treatment can exacerbate existing mental health vulnerabilities or trigger new ones. The physical toll of treatments, ancillary tests (such as blood draws, imaging studies, and numerous cancer/treatment-based tests), changes in appearance, disruptions to normal routines, and extended hospitalizations can all contribute to the development or worsening of MHDs. Additionally, the long-term effects of cancer treatments, such as chemotherapy and radiation, affect neurological functioning, potentially contributing to mental health issues (Jibb et al., 2022; Wakefield et al., 2010). However, it is also possible that more frequent interactions with specialty healthcare providers may identify pre-existing MHDs, contributing to an increased prevalence post-diagnosis.

Specific demographic factors were associated with an increased risk of MHD among childhood cancer patients. Age at diagnosis played an important role, as older age was associated with a higher prevalence of both pre- and post-diagnosis MHD, especially mood disorders. Previous studies have indicated that older childhood cancer patients experience greater emotional distress due to increased vulnerability, physical limitations, and feelings of isolation, which could contribute to higher rates of mood disorders post-diagnosis (Ghandour et al., 2019; Saunders et al., 2022; Geoffroy et al., 2018). Females experienced a higher prevalence of pre-diagnosis mood disorders. This finding is consistent with prior studies showing that girls are more likely to experience anxiety disorders in middle childhood and suffer increased depression rates after puberty (Chaplin et al., 2009; Altemus et al., 2014). Male sex was associated with a greater prevalence of neurodevelopmental disorders in our study, which was also consistent with prior studies (Bölte et al., 2023; Breach and Lenz, 2023; Straub et al., 2022). Variations in prevalence across patient groups emphasize the importance of considering demographic factors when developing tailored mental health interventions for childhood cancer survivors. Certain cancer sites, such as malignant bone tumors, were found to have higher rates of post-diagnosis mood disorders. The finding aligns with a French study showing that adults who survived pediatric bone tumors had significantly higher rates of mood disorders (47.6%) compared to the general population, likely due to the physical pain, loss of mobility, and emotional burden associated with bone tumors (Tardy et al., 2022).

Diagnosis in a more recent year was also associated with a higher prevalence of post-diagnosis MHD and mood disorder in our study. Two potential explanations for this trend are, first, increased awareness and improved screening protocols for mental health in recent years, which may have led to higher detection rates. Second, worsening mental health has been observed among children and teenagers in recent years. During 2013 and 2019, data indicate that 9.4%−9.8% of U.S. children aged 3–17 years were diagnosed with anxiety disorders, which were also the most prevalent MHD. Furthermore, about 20.9% of adolescents aged 12–17 years experienced a major depressive episode (Bitsko et al., 2023). According to a U.S. Health and Human Services study, between 2016 and 2020, the number of children aged 3–17 diagnosed with anxiety increased by 29%, while those diagnosed with depression increased by 27% in the U.S. (Lebrun-Harris et al., 2022).

Of note, among childhood cancer patients diagnosed between 2001 and 2017, those diagnosed from 2014 onward accounted for approximately 33% of the study cohort. This aligns with the Medicaid expansion in 2014, as Kentucky was among the first states to participate in the expansion under the Affordable Care Act. The period from 2014 to 2017 also showed an increase in documented MHD diagnoses, which may reflect improved access to psychosocial services following the Medicaid expansion. These findings suggest a potential association between policy changes and increased identification of mental health needs in this vulnerable population.

Interestingly, socioeconomic factors such as poverty, place of residence, and educational attainment were associated with neurodevelopmental diagnosis. Survivors residing in areas with lower poverty rates were observed to have a higher prevalence of neurodevelopmental disorders. The finding is consistent with prior research (AlAlmaei Asiri et al., 2023), which found parents with a higher education attainment had significantly better knowledge of autism symptoms, leading to earlier identification and treatment for their children (AlAlmaei Asiri et al., 2023). It is plausible that patients in the more impoverished regions were less likely to have neurodevelopmental disorders recognized as a result of less awareness, lower availability of diagnostic resources, and reduced access to healthcare providers. Children in lower-income areas may be underdiagnosed due to limited access to healthcare and a lack of awareness among parents and educators (Andrilla et al., 2018; Yang et al., 2022). Although prior studies have suggested an association between socioeconomic factors and MHDs, particularly mood disorders (Bemis et al., 2015), no such association was observed in our study between socioeconomic factors and either overall MHDs or mood disorder diagnoses. In addition, the relatively homogenous socioeconomic status within our Medicaid-enrolled cohort may not provide sufficient variability to detect such differences. Studies that compare populations across different insurance types or broader socioeconomic strata may be more useful to identify possible associations.

This study has several limitations. First, the use of administrative claims data to identify MHD diagnoses may result in misclassification or underreporting of MHDs. Patients with MHDs might be incorrectly classified into the no-MHD group if they did not receive a formal clinical assessment and, therefore, were not given an MHD diagnosis. Second, our analysis is limited to data from a single state, leading to a smaller sample size that may not be representative of broader populations; thus, larger, population-based studies are needed to better understand the prevalence of MHDs, such as those conducted across the United States. Third, to ensure the quality of the data, the study included only patients who were enrolled in Medicaid for the 12 months before and after their cancer diagnosis. This selection study population may be different from the broader population of Medicaid-enrolled childhood cancer patients. However, this criterion was necessary to ensure complete and consistent data to capture patients' depression diagnosis, enhancing the validity of the findings within the study's scope. Additionally, the study's observational design precludes causal inferences regarding the relationship between cancer diagnosis and the emergence of mental health issues.

More research is needed using prospective study designs with detailed clinical assessments of mental health to explore these associations more thoroughly. Furthermore, investigating the neuropsychological of specific cancer treatments, particularly the neurotoxic effects of specific therapies, could provide insights into their contribution to mental health outcomes. With the high prevalence of MHD in childhood cancer patients, the need for psychosocial service is evident, yet service utilization among children remains insufficient (Whitney and Peterson, 2019). Future research examining mental health-related visits among childhood cancer patients enrolled in Medicaid is essential to better understand benefits of these services within this socioeconomically disadvantaged population.

In conclusion, this study provides information on the prevalence and factors associated with MHDs among childhood cancer patients enrolled in Medicaid in Kentucky. The findings underscore a significant increase in MHD diagnoses following a cancer diagnosis, particularly for mood and neurodevelopmental disorders. Specific demographic factors, including age, gender, socioeconomic status, and cancer type, were found to be associated with the prevalence of MHDs. These results highlight the need for targeted mental health interventions that consider the unique challenges faced by underserved populations, emphasizing the importance of comprehensive mental health support throughout the cancer treatment process to survivorship.

## Data Availability

The data analyzed in this study is subject to the following licenses/restrictions: Due to the agreement between Kentucky Cancer Registry and Kentucky Medicaid Program, we can't share the data. Requests to access these datasets should be directed to Bin Huang, bhuan0@uky.edu.
