# Supplementary material for: Prevalence and characteristics for mental health disorder before and after childhood cancer diagnosis—a statewide population-based study among Medicaid beneficiaries
Source: Front Psychol. 2026 Feb 4;16:1680382. doi: 10.3389/fpsyg.2025.1680382 (PMC12913496; doi:10.3389/fpsyg.2025.1680382)
Supplement: Supplementary file 1 [file Table_1.docx]

Supplementary Material

1. ***Supplementary Figures and Tables***

**Table S1. Classification of 30 Mental Health Disorder (MHD) Groups from the Child and Adolescent Mental Health Disorders Classification System (CAMHD-CS) into Broader MHD Groups with Corresponding ICD-9 and ICD-10 Codes.**

| **Broader Mental Health Disorder Groups** | **Child And Adolescent Mental Health Disorders Classification System (CAMHD-CS)** | **ICD-9 Codes^*^** | **ICD-10 codes^*^** |
| --- | --- | --- | --- |
| Psychotic Disorders | Neurocognitive Disorders | 797, 2900, 2903, 2908–2909, 2930–2931, 2939–2941, 2948–2949, 3100–3102, 3108–3109, 3308, 3310–3312, 3317, 29010–29013, 29020–29021, 29040–29043, 29381–29384, 29389, 29410–29411, 29420–29421, 31081, 33111, 33119, 33182 | F04–F05, F062, F064, F068, F0150–F0151, F0280–F0281, F0390–F0391, F482, F0630–F0634, F0781, F0789, F842, G300–G301, G308–G309, G311, G3101, G3109, G3183, R4181 |
|  | Schizophrenia Spectrum and Other Psychotic Disorders | 2970–2973, 2978–2981, 2983–2984, 2988–2989, 29500–29505, 29510–29515, 29520–29525, 29530–29535, 29540–29545, 29550–29555, 29560–29565, 29570–29575, 29580–29585, 29590–29595 | F060–F061, F200–F203, F205, F2081, F2089, F209, F22–F24, F250–F251, F258–F259, F29 |
| Mood Disorder | Anxiety Disorders | 30000–30002, 30009–30010, 30020–30023, 30029, 30921, 31323 | F408–F411, F413, F418–F419, F930, F940, F4000–F4002, F4010–F4011, F40210, F40218, F40220, F40228, F40230–F40233, F40240–F40243, F40248, F40290–F40291, F40298 |
|  | Bipolar And Related Disorders | 2967, 29600–29604, 29610–29614, 29640–29644, 29650–29654, 29660–29664, 29680–29681, 29689, 30113 | F302–F304, F308–F310, F312, F314–F315, F319, F340, F3010–F3013, F3110–F3113, F3130–F3132, F3160–F3164, F3170–F3178, F3181, F3189 |
|  | Depressive Disorders | 311, 3004, 29620–29624, 29630–29634, 29682 | F320–F325, F328–F333, F3340–F3342, F338–F339, F341, F348, F3481, F3289 |
|  | Dissociative Disorders | 3006, 30012–30015 | F440–F442, F4481, F481 |
|  | Obsessive-Compulsive And Related Disorders | 3003 | F42, F422–F424, F428–F429, F633, F4522 |
|  | Maternal Mental Illness or Substance Abuse During Preg, Delivery or Post Partum | 64830–64834, 64840–64844 | F53, F530–F531, O906, O99310, O99315, O99320–O99325, O99340–O99345 |
|  | Sleep-Wake Disorders | 30740–30749 | F5101–F5105, F5109, F5111–F5113, F5119, F513–F515, F518–F519 |
|  | Somatic Symptom and Related Disorders | 316, 3007, 3060–3064, 3066–3069, 30011, 30016, 30019, 30081–30082, 30650–30653, 30659, 30753–30754, 30780, 30789 | F444–F447, F450–F451, F458–F459, F4520–F4521, F4529, F4541–F4542, F6810–F6813, F68A |
|  | Suicide Or Self-Injury | 9-E954, 9-E956, 9-E959, 9-E9500–E9511, 9-E9518, 9-E9520–E9521, 9-E9528–E9531, 9-E9538–E9539, 9-E9550–E9557, 9-E9559, 9-E9570–E9572, 9-E9579–E9589, V6284 | R45851, T1491(/XA/XS), T360X2A–T364X2A, T360X2S–T364X2S, T365X2A–T368X2A, T365X2S–T368X2S, T3692XA/XS, T370X2A–T374X2A, T370X2S–T374X2S, T375X2A/S, T378X2A/S, T3792XA/XS, T380X2A–T387X2A, T380X2S–T387X2S, T38802A/S, T38812A/S, T38892A/S, T38902A/S, T38992A/S, T39012A/S, T39092A/S, T391X2A/S, T392X2A/S, T39312A/S, T39392A/S, T394X2A/S, T398X2A/S, T3992XA/XS, T400X2A–T408X2A, T400X2S–T408X2S, T40902A/S, T40992A/S, T410X2A/S, T411X2A/S, T41202A/S, T41292A/S, T413X2A/S, T4142XA/XS, T415X2A/S, T420X2A–T428X2A, T420X2S–T428X2S, T43012A/S, T43022A/S, T431X2A/S, T43202A/S, T43212A/S, T43222A/S, T43292A/S, T433X2A/S, T434X2A/S, T43502A/S, T43592A/S, T43602A–T43642A, T43602S–T43642S, T43692A/S, T438X2A/S, T4392XA/XS, T440X2A–T448X2A, T440X2S–T448X2S, T44902A/S, T44992A/S, T450X2A–T454X2A, T450X2S–T454X2S, T45512A/S, T45522A/S, T45602A/S, T45612A/S, T45622A/S, T45692A/S, T457X2A/S, T458X2A/S, T4592XA/XS, T460X2A–T468X2A, T460X2S–T468X2S, T46902A/S, T46992A/S, T470X2A–T478X2A, T470X2S–T478X2S, T4792XA/XS, T480X2A–T481X2A, T480X2S–T481X2S, T48202A/S, T48292A/S, T483X2A/S, T484X2A/S, T485X2A/S, T486X2A/S, T48902A/S, T48992A/S, T490X2A–T494X2A, T490X2S–T494X2S, T495X2A/S, T496X2A/S, T497X2A/S, T498X2A/S, T4992XA/XS, T500X2A–T508X2A, T500X2S–T508X2S, T50902A/S, T50912A/S, T50992A/S, T50A12A/S, T50A22A/S, T50A92A/S, T50B12A/S, T50B92A/S, T50Z12A/S, T50Z92A/S, T510X2A–T511X2A, T510X2S–T511X2S, T512X2A/S, T513X2A/S, T518X2A/S, T5192XA/XS, T520X2A–T524X2A, T520X2S–T524X2S, T528X2A/S, T5292XA/XS, T530X2A–T537X2A, T530X2S–T537X2S, T5392XA/XS, T540X2A–T543X2A, T540X2S–T543X2S, T5492XA/XS, T550X2A/S, T551X2A/S, T560X2A–T567X2A, T560X2S–T567X2S, T56812A/S, T56892A/S, T5692XA/XS, T570X2A–T573X2A, T570X2S–T573X2S, T578X2A/S, T5792XA/XS, T5802XA/S, T5812XA, T582X2A/S, T588X2A/S, T5892XA/XS, T590X2A–T597X2A, T590X2S–T597X2S, T59812A/S, T59892A/S, T5992XA/XS, T600X2A–T604X2A, T600X2S–T604X2S, T608X2A/S, T6092XA/XS, T6102XA/XS, T6112XA/XS, T61772A/S, T61782A/S, T618X2A/S, T6192XA/XS, T620X2A/S, T621X2A/S, T622X2A/S, T628X2A/S, T6292XA/XS, T63002A–T63092A, T63002S–T63092S, T63112A/S, T63122A/S, T63192A/S, T632X2A/S, T63302A–T63332A, T63302S–T63332S, T63392A/S, T63412A–T63442A, T63412S–T63442S, T63452A/S, T63462A/S, T63482A/S, T63512A/S, T63592A/S, T63612A–T63632A, T63612S–T63632S, T63692A/S, T63712A/S, T63792A/S, T63812A–T63832A, T63812S–T63832S, T63892A/S, T6392XA/XS, T6402XA/XS, T6482XA/XS, T650X2A/S, T651X2A/S, T65212A/S, T65222A/S, T65292A/S, T653X2A/S, T654X2A/S, T655X2A/S, T656X2A/S, T65812A–T65832A, T65812S–T65832S, T65892A/S, T6592XA/XS, T71112A/S, T71122A/S, T71132A/S, T71152A/S, T71162A/S, T71192A/S, T71222A/S, T71232A/S, X710XX(A/D/S)–X713XX(A/D/S), X718XX(A/D/S), X719XX(A/D/S), X72XXX(A/D/S), X730XX(A/D/S)–X732XX(A/D/S), X738XX(A/D/S), X739XX(A/D/S), X7401XA–X7402XA, X7401XD–X7402XD, X7401XS–X7402XS, X7409XA/XD/XS, X748XX(A/D/S), X749XX(A/D/S), X75XXX(A/D/S), X76XXX(A/D/S), X770XX(A/D/S)–X773XX(A/D/S), X778XX(A/D/S), X779XX(A/D/S), X780XX(A/D/S), X781XX(A/D/S), X782XX(A/D/S), X788XX(A/D/S), X789XX(A/D/S), X79XXX(A/D/S), X80XXX(A/D/S), X810XX(A/D/S), X811XX(A/D/S), X818XX(A/D/S), X820XX(A/D/S), X821XX(A/D/S), X822XX(A/D/S), X828XX(A/D/S), X830XX(A/D/S), X831XX(A/D/S), X832XX(A/D/S), X838XX(A/D/S) |
|  | Trauma And Stressor-Related Disorders | 298, 308-309 | F430-F432, F438-F439, F941-F942 |
| Personality Disorder | Personality Disorders | 3010, 3013–3014, 3016–3017, 3019, 30110–30112, 30120–30122, 30150–30151, 30159, 30181–30184, 30189 | F21, F600–F607, F609, F6081, F6089 |
|  | Sexuality And Gender Identity Disorders | 3020–3024, 3026, 3029, 30250–30253, 30270–30276, 30279, 30281–30285, 30289, 31382 | F66, F520–F521, F524–F526, F528–F529, F640–F642, F648–F654, F659, F5221–F5222, F5231–F5232, F6550–F6552, F6581, F6589, R37, Z87890 |
| Substance Use Disorders | Substance Abuse-Related Medical Illness | 3575, 4255, 5353, 5710–5713, 53530–53531 | G312, G621, I426, K700, K702, K709, K2920–K2921, K7010–K7011, K7030–K7031, K7040–K7041, O99311–O99314, R781–R785 |
|  | Substance-Related and Addictive Disorders | 2910–2915, 2918–2920, 2922, 2929, 3051, 29181–29182, 29189, 29211–29212, 29281–29285, 29289, 30300–30303, 30390–30393, 30400–30403, 30410–30413, 30420–30423, 30430–30433, 30440–30443, 30450–30453, 30460–30463, 30470–30473, 30480–30483, 30490–30493, 30500–30503, 30510–30513, 30520–30523, 30530–30533, 30540–30543, 30550–30553, 30560–30563, 30570–30573, 30580–30583, 30590–30593 | F1010, F1113, F1213, F1413, F1493, F1513, F10120–F10121, F10129–F10132, F10139, F10930–F10932, F10939, F13130–F13132, F13139, F19130–F19132, F19139 |
| Neuropsychiatric/ Developmental Disorders | ADHD | 3140–3142, 3148–3149 | F900-F902, F908-F909 |
|  | Autism Spectrum Disorder | 29900–29901, 29910–29911, 29980–29981, 29990–29991 | F840, F843, F845, F848-F849 |
|  | Communication Disorders | 3070, 31534–31535, 31539, V401 | F800, F804, F809, F985, F8081–F8082, F8089 |
|  | Developmental Delay or Unspecified Neurodevelopmental Disorder | 3155, 3158–3159 | F88–F89, F819, F8189 |
|  | Disruptive, Impulse Control and Conduct Disorders | 3124, 3128–3129, 31200–31203, 31210–31213, 31220–31223, 31230–31235, 31239, 31281–31282, 31289, 31381 | F630–F632, F639, F910–F913, F918–F919, F6381, F6389 |
|  | Elimination Disorders | 3076–3077, 78760–78763 | F980-F981, R150-R152, R159 |
|  | Fetal Or Newborn Damage Related to Maternal Substance Abuse | 7795, 7903, 9800, 65540–65541, 65543, 65550–65551, 65553, 76071–76073, 76075 | O354XX0–O354XX5, O354XX9–O355XX5, O355XX9, P0414, P0416–P0417, P043, P0440–P0442, P0449, P0481, P961, Q860, R780 |
|  | Intellectual Disability | 317, 319, 3180–3182 | F70-F73, F78-F79, R4183 |
|  | Motor Disorders | 3073, 3154, 30720–30723 | F82, F950–F952, F958–F959, F984 |
|  | Specific Learning Disorders | 3151–3152, 31500–31502, 31509, 31531–31532, V400 | F801–F802, F810, F812, F8181, H9325, R480 |
| Eating Disorder | Feeding And Eating Disorders | 3071, 30750–30752, 30759 | F502, F508–F509, F983, F5000–F5002, F5081–F5082, F5089, F9821, F9829 |
| Other MHD | Mental Health Symptom | 3079, 3089, 7801, 78095, 7992, 79921–79925, 79929, 79951–79955, 79959, V402, V4031, V409, V6285, V7102 | F59, R440–R443, R450–R457, R4581–R4584, R45850, R4586–R4587, R4589, R460–R467, R4681, Z72810, Z9183 |
|  | Miscellaneous | 3005, 3009, 3131, 3133, 3139, 6254, 29690, 29699, 30089, 30922–30923, 31089, 31321–31322, 31383, 31389, 79099, V6289 | F09, F28, F39, F54, F69–F070, F079, F99, F349, F449, F488–F489, F550–F554, F558, F688, F938–F939, F948–F949, F988–F989, F3281, F3489, F4489, R419, R4189, R41840–R41844 |
|  | Accidental Or Undetermined Poisoning | 9696, 96500–96502, 96509, 97081 | T40411A, T40414A, T40421A, T40424A, T40491A, T40494A, T40601A, T40604A, T40691A, T40694A, T40901A, T40904A, T40991A, T40994A, T43641A, T43644A, T40411S, T40414S, T40421S, T40424S, T40491S, T40494S, T43641S, T43644S, T400X1A, T400X4A, T401X1A, T401X4A, T402X1A, T402X4A, T403X1A, T403X4A, T404X1A, T404X4A, T405X1A, T405X4A, T407X1A, T407X4A, T408X1A, T408X4A, T426X4A, T510X1A, T510X4A |

*** ICD-9 and ICD-10 Codes are summarized from the Child and Adolescent Mental Health Disorders Classification System**

**Table S2. Characteristics of Medicaid-enrolled Childhood Cancer Patients Diagnosed from 2001 to 2017 in Kentucky stratified by Pre-Diagnosis Mood Disorder Status.**

| **Characteristic** | **Category** | **Total** | **With Pre-Diagnosis Mood Disorder** | | **p-value** | **With Post-Diagnosis Mood Disorder** | |  |
| --- | --- | --- | --- | --- | --- | --- | --- | --- |
|  |  |  | **No (n=758)** | **Yes (n=120)** | **(2 sided)** | **No (n=667)** | **Yes (n=311)** | **p-value** |
|  |  |  | **N (%)** | **N(%)** |  | **N (%)** | **N(%)** | **(2 sided)** |
|  | | | | | |  | | |
| Age Group | 0-4 | 306 | 300(98) | 6(2) | <0.0001 | 256(83.7) | 50(16.3) | <0.0001 |
|  | 5-9 | 212 | 194(91.5) | 18(8.5) |  | 148(69.8) | 64(30.2) |  |
|  | 10-14 | 217 | 188(86.6) | 29(13.4) |  | 134(61.8) | 83(38.2) |  |
|  | 15-19 | 243 | 176(72.4) | 67(27.6) |  | 129(53.1) | 114(46.9) |  |
|  | | | | | |  | | |
| Gender | Male | 528 | 476(90.2) | 52(9.8) | 0.0124 | 367(69.5) | 161(30.5) | 0.3417 |
|  | Female | 450 | 382(84.9) | 68(15.1) |  | 300(66.7) | 150(33.3) |  |
|  | | | | | |  | | |
| Race | White | 831 | 724(87.1) | 107(12.9) | 0.3512 | 551(66.3) | 280(33.7) | 0.0064 |
|  | Black | 129 | 117(90.7) | 12(9.3) |  | 100(77.5) | 29(22.5) |  |
|  | Other | 18 | 17(94.4) | 1(5.6) |  | 16(88.9) | 2(11.1) |  |
|  | | | | | |  | | |
| Metro Status | Non-metro | 534 | 466(87.3) | 68(12.7) | 0.6275 | 369(69.1) | 165(30.9) | 0.5071 |
|  | Metro | 444 | 392(88.3) | 52(11.7) |  | 298(67.1) | 146(32.9) |  |
|  | | | | | |  | | |
| Appalachian Status | Non-Appalachia | 593 | 531(89.5) | 62(10.5) | 0.0318 | 407(68.6) | 186(31.4) | 0.7178 |
|  | Appalachia | 385 | 327(84.9) | 58(15.1) |  | 260(67.5) | 125(32.5) |  |
|  | | | | | |  | | |
| % high school or higher education | 0-73.16% | 251 | 213(84.9) | 38(15.1) | 0.4387 | 173(68.9) | 78(31.1) | 0.9711 |
|  | 73.17%-81.86% | 244 | 215(88.1) | 29(11.9) |  | 168(68.9) | 76(31.1) |  |
|  | 81.97%-87.95% | 247 | 220(89.1) | 27(10.9) |  | 166(67.2) | 81(32.8) |  |
|  | >=87.96% | 236 | 210(89) | 26(11) |  | 160(67.8) | 76(32.2) |  |
|  | | | | | |  | | |
| % below poverty 2010 | 0-16.45% | 360 | 319(88.6) | 41(11.4) | 0.9263 | 245(68.1) | 115(31.9) | 0.2948 |
|  | 16.46%-18.91% | 147 | 128(87.1) | 19(12.9) |  | 91(61.9) | 56(38.1) |  |
|  | 18.92%-23.67% | 234 | 205(87.6) | 29(12.4) |  | 163(69.7) | 71(30.3) |  |
|  | >=23.68% | 237 | 206(86.9) | 31(13.1) |  | 168(70.9) | 69(29.1) |  |
|  | | | | | |  | | |
| Year of Diagnosis | 2001-2005 | 198 | 179(90.4) | 19(9.6) | 0.3533 | 152(76.8) | 46(23.2) | <0.0001 |
|  | 2006-2009 | 192 | 165(85.9) | 27(14.1) |  | 134(69.8) | 58(30.2) |  |
|  | 2010-2013 | 258 | 221(85.7) | 37(14.3) |  | 186(72.1) | 72(27.9) |  |
|  | 2014-2017 | 330 | 293(88.8) | 37(11.2) |  | 195(59.1) | 135(40.9) |  |
|  | | | | | |  | | |
| ICCC Site | Leukemias, Myeloproliferative and Myelodysplastic Diseases | 238 | 212(89.1) | 26(10.9) | 0.0062 | 157(66) | 81(34) | <0.0001 |
|  | Lymphomas and reticuloendothelial neoplasms | 147 | 133(90.5) | 14(9.5) |  | 94(63.9) | 53(36.1) |  |
|  | CNS and Miscellaneous Intracranial and Intraspinal Neoplasms | 220 | 191(86.8) | 29(13.2) |  | 161(73.2) | 59(26.8) |  |
|  | Neuroblastoma And Other Peripheral Nervous Cell Tumors | 50 | 48(96) | 2(4) |  | 42(84) | 8(16) |  |
|  | Retinoblastoma | 14 | 14(100) | 0(0) |  | 13(92.9) | 1(7.1) |  |
|  | Renal Tumors | 33 | 33(100) | 0(0) |  | 28(84.8) | 5(15.2) |  |
|  | Hepatic Tumors | 11 | 10(90.9) | 1(9.1) |  | 9(81.8) | 2(18.2) |  |
|  | Malignant Bone Tumors | 58 | 49(84.5) | 9(15.5) |  | 21(36.2) | 37(63.8) |  |
|  | Soft Tissue and Other Extraosseous Sarcomas | 55 | 49(89.1) | 6(10.9) |  | 39(70.9) | 16(29.1) |  |
|  | Germ Cell Tumors, Trophoblastic Tumors and Neoplasms of Gonads | 49 | 37(75.5) | 12(24.5) |  | 32(65.3) | 17(34.7) |  |
|  | Other Malignant Epithelial Neoplasms and Malignant Melanomas | 90 | 73(81.1) | 17(18.9) |  | 60(66.7) | 30(33.3) |  |
|  | Other And Unspecified Malignant Neoplasms | 1 | 1(100) | 0(0) |  | 1(100) | 0(0) |  |
|  | Unknown | 12 | 8(66.7) | 4(33.3) |  | 10(83.3) | 2(16.7) |  |

**Table S3. Multivariable Logistic Regression Analysis for Identifying Risks Factors for Pre- and Post-Diagnosis Mood Disorder**

|  | **Pre-Diagnosis Mood Disorder** | | **Post-Diagnosis Mood Disorder** | |
| --- | --- | --- | --- | --- |
| **Effect** | **Odds Ratio** | **95% CI** | **Odds Ratio** | **95% CI** |
| **Age at Diagnosis (Reference: 0-4)** | - | - | - | - |
| 5-9 | 4.7 | 1.8 - 12.1 | 2.0 | 1.2 - 3.1 |
| 10-14 | 7.6 | 3.1 - 18.8 | 2.6 | 1.6 - 4.2 |
| 15-19 | 18.4 | 7.8 - 43.4 | 3.6 | 2.3 – 6.0 |
| **sex** (Reference: Male) |  | - | - | - |
| Female | 1.6 | 1.0 - 2.3 | - | - |
| **Appalachian Status** (Reference: Non-Appalachian) | - | - | - | - |
| Appalachian | 1.3 | 0.9 - 2.0 | - | - |
| **Race** (Reference: Black) | - | - | - | - |
| Other | - | - | 0.4 | 0.1 – 2.0 |
| White | - | - | 1.7 | 1.0 - 2.8 |
| **Year of Diagnosis** (Reference: 2014-2017) | - | - | - | - |
| 2001-2005 | - | - | 0.4 | 0.3 - 0.6 |
| 2006-2009 | - | - | 0.5 | 0.4 - 0.8 |
| 2010-2013 | - | - | 0.4 | 0.3 - 0.7 |
| **ICCC** **Site** (Reference: Leukemias, Myeloproliferative and Myelodysplastic Diseases) | - | - | - | - |
| CNS and Miscellaneous Intracranial and Intraspinal Neoplasms | - | - | 0.6 | 0.4 – 0.9 |
| Germ Cell Tumors, Trophoblastic Tumors and Neoplasms of Gonads | - | - | 0.4 | 0.2 – 0.9 |
| Hepatic Tumors | - | - | 0.6 | 0.1 – 3.2 |
| Lymphomas and Reticuloendothelial Neoplasms | - | - | 0.8 | 0.5 - 1.2 |
| Malignant Bone Tumors | - | - | 2.5 | 1.3 - 4.9 |
| Neuroblastoma and Other Peripheral Nervous Cell Tumors | - | - | 0.6 | 0.2 - 1.4 |
| Other And Unspecified Malignant Neoplasms | - | - | NA* | NA* |
| Other Malignant Epithelial Neoplasms and Malignant Melanomas | - | - | 0.4 | 0.2 - 0.7 |
| Renal Tumors | - | - | 0.5 | 0.3 - 1.1 |
| Retinoblastoma | - | - | 0.3 | 0.0 - 2.7 |
| Soft Tissue and Other Extraosseous Sarcomas | - | - | 0.5 | 0.3 - 1.1 |
| Unknown | - | - | 0.1 | 0.0 – 0.7 |
| **Pre-diagnosis Mood Disorder (reference: No)** | - | - |  |  |
| Yes | - | - | 5.6 | 3.5-9.0 |

*NA indicates unstable estimates resulting from small sample sizes

**Table S4. Characteristics of Medicaid-enrolled Childhood Cancer Patients Diagnosed from 2001 to 2017 in Kentucky stratified by Pre-Diagnosis Neuropsychiatric/developmental Disorder Status.**

| **Characteristic** | **Category** |  | **With Pre-Diagnosis Neuropsychiatric/developmental Disorder** | | **p-value** | **With Post-Diagnosis Neuropsychiatric/developmental Disorder** | | **p-value** |
| --- | --- | --- | --- | --- | --- | --- | --- | --- |
|  |  | **Total** | **No (n=750)** | **Yes (n=228)** | **(2 sided)** | **No (n=711)** | **Yes (n=267)** | **(2 sided)** |
|  |  |  | **N (%)** | **N (%)** |  | **N (%)** | **N (%)** |  |
|  | | | | | |  | | |
| Age Group | 0-4 | 306 | 249(81.4) | 57(18.6) | 0.0605 | 219(71.6) | 87(28.4) | 0.0454 |
|  | 9-May | 212 | 151(71.2) | 61(28.8) |  | 143(67.5) | 69(32.5) |  |
|  | 14-Oct | 217 | 165(76) | 52(24) |  | 157(72.4) | 60(27.6) |  |
|  | 15-19 | 243 | 185(76.1) | 58(23.9) |  | 192(79) | 51(21) |  |
|  | | | | | |  | | |
| Gender | Male | 528 | 385(72.9) | 143(27.1) | 0.0025 | 359(68) | 169(32) | 0.0003 |
|  | Female | 450 | 365(81.1) | 85(18.9) |  | 352(78.2) | 98(21.8) |  |
|  | | | | | |  | | |
| Race | White | 831 | 641(77.1) | 190(22.9) | 0.4514 | 611(73.5) | 220(26.5) | 0.2363 |
|  | Black | 129 | 94(72.9) | 35(27.1) |  | 86(66.7) | 43(33.3) |  |
|  | Other | 18 | 15(83.3) | 3(16.7) |  | 14(77.8) | 4(22.2) |  |
|  | | | | | |  | | |
| Metro Status | Non-metro | 534 | 425(79.6) | 109(20.4) | 0.0186 | 406(76) | 128(24) | 0.0103 |
|  | Metro | 444 | 325(73.2) | 119(26.8) |  | 305(68.7) | 139(31.3) |  |
|  | | | | | |  | | |
| Appalachian Status | Non-Appalachia | 593 | 438(73.9) | 155(26.1) | 0.0095 | 419(70.7) | 174(29.3) | 0.0753 |
|  | Appalachia | 385 | 312(81) | 73(19) |  | 292(75.8) | 93(24.2) |  |
|  | | | | | |  | | |
| % high school or higher completion | 0-73.16% | 251 | 211(84.1) | 40(15.9) | 0.0064 | 194(77.3) | 57(22.7) | 0.0133 |
|  | 73.17%-81.86% | 244 | 188(77) | 56(23) |  | 183(75) | 61(25) |  |
|  | 81.97%-87.95% | 247 | 182(73.7) | 65(26.3) |  | 181(73.3) | 66(26.7) |  |
|  | >=87.96% | 236 | 169(71.6) | 67(28.4) |  | 153(64.8) | 83(35.2) |  |
|  | | | | | |  | | |
| % below poverty 2010 | 0-16.45% | 360 | 259(71.9) | 101(28.1) | 0.0014 | 246(68.3) | 114(31.7) | 0.0283 |
|  | 16.46%-18.91% | 147 | 107(72.8) | 40(27.2) |  | 103(70.1) | 44(29.9) |  |
|  | 18.92%-23.67% | 234 | 182(77.8) | 52(22.2) |  | 175(74.8) | 59(25.2) |  |
|  | >=23.68% | 237 | 202(85.2) | 35(14.8) |  | 187(78.9) | 50(21.1) |  |
|  | | | | | |  | | |
| Year of Diagnosis | 2001-2005 | 198 | 160(80.8) | 38(19.2) | 0.4327 | 160(80.8) | 38(19.2) | 0.0134 |
|  | 2006-2009 | 192 | 147(76.6) | 45(23.4) |  | 128(66.7) | 64(33.3) |  |
|  | 2010-2013 | 258 | 197(76.4) | 61(23.6) |  | 182(70.5) | 76(29.5) |  |
|  | 2014-2017 | 330 | 246(74.5) | 84(25.5) |  | 241(73) | 89(27) |  |
|  | | | | | |  | | |
| ICCC Site | Leukemias, Myeloproliferative and Myelodysplastic Diseases | 238 | 188(79) | 50(21) | 0.5155 | 177(74.4) | 61(25.6) | 0.4793 |
|  | Lymphomas and reticuloendothelial neoplasms | 147 | 115(78.2) | 32(21.8) |  | 108(73.5) | 39(26.5) |  |
|  | CNS and Miscellaneous Intracranial and Intraspinal Neoplasms | 220 | 156(70.9) | 64(29.1) |  | 149(67.7) | 71(32.3) |  |
|  | Neuroblastoma And Other Peripheral Nervous Cell Tumors | 50 | 37(74) | 13(26) |  | 32(64) | 18(36) |  |
|  | Retinoblastoma | 14 | 12(85.7) | 2(14.3) |  | 11(78.6) | 3(21.4) |  |
|  | Renal Tumors | 33 | 27(81.8) | 6(18.2) |  | 26(78.8) | 7(21.2) |  |
|  | Hepatic Tumors | 11 | 8(72.7) | 3(27.3) |  | 7(63.6) | 4(36.4) |  |
|  | Malignant Bone Tumors | 58 | 44(75.9) | 14(24.1) |  | 43(74.1) | 15(25.9) |  |
|  | Soft Tissue and Other Extraosseous Sarcomas | 55 | 47(85.5) | 8(14.5) |  | 42(76.4) | 13(23.6) |  |
|  | Germ Cell Tumors, Trophoblastic Tumors and Neoplasms of Gonads | 49 | 34(69.4) | 15(30.6) |  | 37(75.5) | 12(24.5) |  |
|  | Other Malignant Epithelial Neoplasms and Malignant Melanomas | 90 | 72(80) | 18(20) |  | 71(78.9) | 19(21.1) |  |
|  | Other And Unspecified Malignant Neoplasms | 1 | 1(100) | 0(0) |  | 0(0) | 1(100) |  |
|  | Unknown | 12 | 9(75) | 3(25) |  | 8(66.7) | 4(33.3) |  |

**Table S5. Multivariable Logistic Regression Analysis for Identifying Risks Factors for Pre- and Post-Diagnosis Mood Disorder**

|  | **Pre-Diagnosis Neuropsychiatric/developmental Disorder** | | **Post-Diagnosis Neuropsychiatric/developmental Disorder** | |
| --- | --- | --- | --- | --- |
| **Effect** | **Odds Ratio** | **95% CI** | **Odds Ratio** | **95% CI** |
| **Age at Diagnosis (Reference: 0-4)** | **-** | **-** |  |  |
| 5-9 | **-** | **-** | 0.8 | 0.5-1.3 |
| 10-14 | **-** | **-** | 0.7 | 0.5-1.2 |
| 15-19 | **-** | **-** | 0.5 | 0.3-0.7 |
| **Sex** (Reference: Female) | - | - | - | - |
| Male | 1.6 | 1.3 – 2.0 | 1.5 | 1.1 – 2.1 |
| **% below poverty 2010** (Reference: >=23.68%) | - | - | - | - |
| 0-16.45% | 2.3 | 1.5 - 3.5 | - | - |
| 16.46%-18.91% | 2.3 | 1.4 - 3.8 | - | - |
| 18.92%-23.67% | 1.7 | 1.0 - 2.7 | - | - |
| **Year of Diagnosis** (Reference: 2014-2017) | - | - | - | - |
| 2001-2005 | - | - | 0.7 | 0.4 - 1.1 |
| 2006-2009 | - | - | 1.6 | 1.0 - 2.6 |
| 2010-2013 | - | - | 1.2 | 0.8 - 1.9 |
| **Pre-diagnosis** **Neuropsychiatric/developmental Disorder (reference= “No”)** |  |  |  |  |
| Yes |  |  | 15.2 | 10.5-21.9 |
